# Spotted Fever Group Rickettsiae in Ticks, Germany

**DOI:** 10.3201/eid1705.101445

**Published:** 2011-05

**Authors:** Cornelia Silaghi, Dietmar Hamel, Claudia Thiel, Kurt Pfister, Martin Pfeffer

**Affiliations:** Author affiliations: Ludwig-Maximilians-Universität, Munich, Germany (C. Silaghi, D. Hamel, C. Thiel, K. Pfister);; Universität Leipzig, Leipzig, Germany (M. Pfeffer)

**Keywords:** Rickettsia, vector-borne infections, bacteria, ticks Germany, dispatch

## Abstract

To explore increased risk for human *Rickettsia* spp. infection in Germany, we investigated recreational areas and renatured brown coal surface-mining sites (also used for recreation) for the presence of spotted fever group rickettsiae in ticks. *R*. *raoultii* (56.7%), *R*. *slovaca* (13.3%), and *R*. *helvetica* (>13.4%) were detected in the respective tick species.

*Rickettsia* species of the spotted fever group are causing emerging infectious diseases (*1*). Since 1977, *Rickettsia slovaca*, found in *Dermacentor marginatus* ticks, was the only known *Rickettsia* sp. in Germany until 2002, when the following were identified: *R. monacensis* and *R. helvetica* in *Ixodes ricinus* ticks, *Rickettsia* sp. RpA4 (now *R. raoultii*) in *D. reticulatus* ticks, *R. felis* in *Ctenocephalides felis* cat fleas, and *R. massiliae* in *I. ricinus* ticks (*1**,**2*). All of these species cause tick-borne rickettsioses in humans, including tick-borne lymphadenopathy (TIBOLA) (*3**–**7*). The aim of this study was to explore the interface between the vector tick and humans by investigating the presence of *Rickettsia* spp. in ticks at highly frequented recreational areas and renatured brown coal surface-mining sites that also are used for leisure.

## The Study

Questing ticks were collected from vegetation by flagging in 3 regions in Germany (9 sites total) in March–September 2008 and April–October 2009 (Figure). Three sites, including renatured gravel pits and walking areas near villages and cities (A–C), were located in the federal state of Saarland. One site in southern Germany was in a natural alluvial forest north of Munich, popular for hiking and dog walking (D), and in East Germany (Saxony), 3 sites were former brown coal surface-mining areas near the city of Leipzig (E–G). Ongoing renaturation and flooding of the pit holes during past decades created a highly valuable recreational area with artificial lakes and surrounding meadows and forest (www.leipzigerneuseenland.de). Here, sampling was carried out around Lake Cospuden (436 ha, fully flooded for the past 10 years, 51.5 m deep; Figure). Further sampling sites were located on a renatured former waste disposal area (H), now a popular urban recreation area, and in an alluvial forest near a popular game park (I), both within Leipzig.

**Figure:**
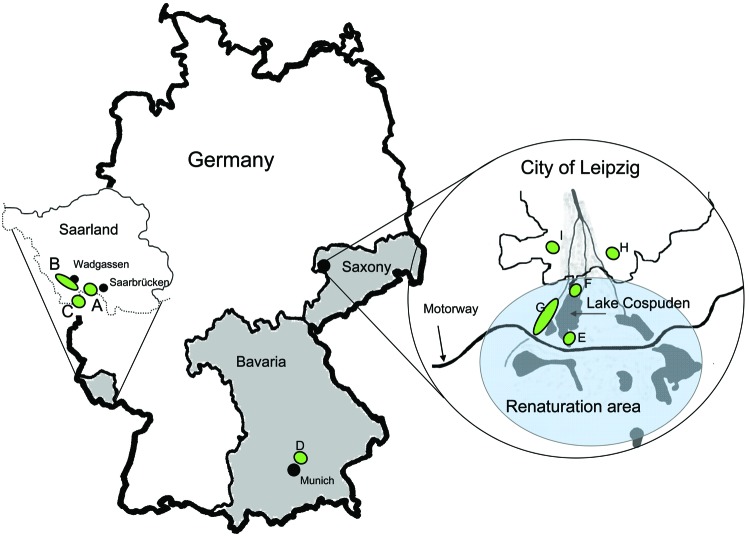
Tick sampling sites in Germany during 2008 and 2009 (green shading): Saarland, sites A–C, along the border with France (sampled March–September 2008). Bavaria, site D (sampled April and September 2009). Saxony, sites E–G, in Lake Cospuden renatured brown coal surface-mining area (blue shading); site H, renatured waste disposal site (sampled April and September/October 2009); and site I, game park (sampled in April 2009). Federal states of Saarland, Bavaria, and Saxony are shaded in gray.

Ticks were stored either in 70% ethanol or kept at 4°C, identified to species level, and homogenized (ticks in ethanol: 2 mL Eppendorf tubes [Eppendorf, Hamburg, Germany] with one 5-mm steel bead, 80 µL phosphate-buffered saline, and in Tissue Lyser [QIAGEN, Hilden, Germany] for 5 min at 30.000U/min; live ticks: 2.0 mp PRECELLYS Kit Mk28, 320 µL minimum essential medium cell culture medium [Sigma-Aldrich, Taufkirchen, Germany], in a PRECELLYS 24 dual [Peqlab, Erlangen, Germany] for 2 × 5,000 rpm for 30 s, with a 15-s break). DNA was extracted from each tick separately (females, males, nymphs) or in pools of 5 individuals (nymphs only) with the QIAGEN DNA Mini Kit (QIAGEN) by using the protocol for animal tissue. Quality and quantity of extracted DNA were tested with a NANO DROP ND-1000 spectrophotometer (Peqlab).

Ticks were screened for rickettsial DNA by using a PCR amplifying part of the *gltA* gene as described (*8*). Some tick samples positive for *Rickettsia* spp. were selected to amplify and determine the sequence of the outer membrane protein–coding genes *ompA* and *ompB* (*9*). New primers were developed for the first part of the *ompA* gene: OmpA-MMX1-for 5′-ACAAGCTGGAGGAAGCCTAGC-3′; OmpA-MMX1-rev 5′-TCTCCCGCTCCTTTGAAAACTAT-3′.

Of the 3,076 ticks collected, 1,359 were identified as *D. reticulatus* ticks (542 males, 817 females, from all sites except site I), 15 as *D. marginatus* ticks (8 males, 7 females, only at site B), and 1,702 as *I. ricinus* ticks (714 males, 658 females, 330 nymphs, from all sites). DNA was extracted from most *D. reticulatus* ticks and almost half of the *I. ricinus* ticks (Table). In the *gltA* PCR, 849 bands of the correct size were obtained in the investigated samples (Table; 749/1,322 (56.7%) of *D. reticulatus* ticks; 2/15 (13.3 %) of *D. marginatus* ticks; 98/730 (13.4%–17.4%) of *I. ricinus* ticks (772 nymphs in pools of up to 5). The rate of infection differed by tick species, sex of the tick, and collection site; for example, the infection rate was 20.5%–76.4% in female *D. reticulatus* ticks (Table). Sequencing of 24.1% (n = 205) of the *gltA* PCR-products verified the specific amplification of rickettsial DNA in 192 cases (93.6%); sequencing for the remaining 13 amplicons could not be determined because the sequencing result was of poor quality. In 1 *I. ricinus* tick from site B, 87%–88% identity to *R. asiatica*, *R. canadensis*, and *R. helvetica* was identified, but amplification of *ompA* and *ompB* failed to further verify the species.

**Table:** *Rickettsia* spp. found in ticks from different sites, Germany, 2008–2009*

State (region)	Site†	No. positive/total no. (%)								
		*Ixodes ricinus* ticks				*Dermacentor reticulatus* ticks			*D. marginatus* ticks	
		M	F	Nymphs‡		M	F		M	F
Saarland (West)	A	1/6 (16.6)	0/6 ( 0)	1–3/8 (12.5–37.5)		5/14 (35.7)	7/14 (50)		NA	NA
	B	8/26 (30.8)	6/35 (17.1)	3–7/14 (21.4–50.0)		67/128 (52.3)	71/153 (46.4)		1/8 (12.5)	1/7 (14.3)
	C	2/4 (50.0)	0/2 (0)	2–7/14 (14.3–50.0)		9/35 (25.7)	8/39 (20.5)		NA	NA
Bavaria (South)	D	11/58 (19.0)	8/42 (19.0)	6/28 (21.4)		11/40 (27.5)	30/95 (31.6)		NA	NA
Saxony (East)	E	5/56 (8.9)	10/45 (22.2)	0/6 (0)		144/190 (75.8)	236/309 (76.4)		NA	NA
	F	1/4 (25.0)	1/6 (16.6)	2–10/10 (20.0–100)		17/21 (80.1)	21/29 (72.4)		NA	NA
	G	0/2 (0)	1/6 (16.6)	1–5/13 (7.7–38.4)		30/80 (37.5)	50/111 (45.0)		NA	NA
	H	8/53 (15.1)	4/74 (5.4)	4–11/35 (11.4–31.4)		10/16 (62.5)	33/48 (68.1)		NA	NA
	I	7/104 (6.7)	5/81 (6.2)	1/34 (2.9)		NA	NA		NA	NA
Total	A–I	43/313 (13.7)	35/297 (11.7)	20–50/162 (12.3–30.1)		293/524 (55.5)	457/798 (57.1)		1/8 (12.5)	1/7 (14.3)

*NA, not available. †For exact location of site, see Figure. ‡In the regions Saarland and Saxony, some of the nymphs were placed in pools of up to 5 specimens; range of positive ticks indicates the possible minimum and maximum number of ticks positive for *Rickettsia* spp. in a pool.

From each site, 1–6 positive tick samples were analyzed for the *ompA* and *ompB* genes. The *ompB* genes of *R. raoultii* (n = 27), *R. helvetica* (n = 8), and *R. slovaca* (n = 2), and *ompA* genes of *R. raoultii* (n = 25) and *R. slovaca* (n = 2) were 100% identical in the amplified part of the respective species, regardless of geographic origin. *R. raoultii* from our study showed 100% identity (*ompA*) and 99% identity (*ompB*) to *R*. *raoultii* strain Marne (GenBank accession nos. DQ365800 and DQ365797); for *R. helvetica* (*ompB*), 100% identity to GenBank entry AF123725; and for *R. slovaca,* 100% identity to GenBank entry AF123723 (*ompB*), and U83454 (*ompA*). *R. raoultii*, *R. slovaca*, and *R. helvetica* were found only in *D. reticulatus*, *D. marginatus*, and *I. ricinus* ticks, respectively. Sequences (*ompA* and *ompB*) from this study were deposited in GenBank (accession nos. HQ232215–HQ232278).

## Conclusions

In Germany, the most common tick is *I. ricinus*; *D. reticulatus* ticks have a focal distribution, and *D. marginatus* ticks have been described on only a few occasions because the latter require warm and dry habitats (*2**,**10**,**11*). Climate change and structural landscape changes have been discussed as reasons for the creation of new tick habitats (*12*). Brown coal surface-mining sites of the former German Democratic Republic undergo extensive renaturation, thus providing new biotopes for many plant and animal species, including ticks.

In Germany, *R. slovaca* was first described in 1977 (*2*) and again recently (*11*). Even though the sample size in the present study was small, comparable prevalence rates were detected (13%). *R. raoultii* was first detected in Russia and has recently been described as a new species (*13*). The average infection rate of *R. raoultii* in this study was 56.7% and, in the renatured brown coal surface-mining sites, ≈80.1%. The latter rate is high in comparison with results of previous studies (*11**,**12*). *R. helvetica* prevalence in *I. ricinus* ticks was similar to results of other studies in Germany (*8*).

Our results confirm the presence of these rickettsial pathogens in Germany. In addition, we identified previously unknown areas where *Rickettsia* spp. are endemic. This finding is of major concern to public health: both *R. slovaca* and *R. raoultii* can cause TIBOLA, even though *R. slovaca* is considered to be more pathogenic (*7**,**14*). A case of TIBOLA caused by *R. slovaca* was identified in a human patient in an *R. slovaca*–endemic area in western Germany (*11*). The pathogenicity of *R. helvetica* has not been fully clarified, but serologic evidence shows human infections in France (*3*), and DNA of *R. helvetica* was recently identified in a patient in Sweden who had meningitis (*15*).

Renaturation of industrial sites specifically provides new areas for human recreation and, simultaneously, new habitats for many plant and animal species. Previously nonexistent opportunities for intensive contact between vector ticks and humans are now available. Thus, human-made habitats may lead to increased emerging diseases, especially in tick-borne rickettsioses, because renaturation areas may form favorable biotopes for enhanced human–vector interactions.
